# Fracture resistance of roots enlarged with various rotary systems and obturated with different sealers

**DOI:** 10.15171/joddd.2019.033

**Published:** 2019-10-07

**Authors:** Selen İnce Yusufoglu, Melek Akman, Makbule Bilge Akbulut, Ayce Ünverdi Eldeniz

**Affiliations:** ^1^Department of Endodontics, Faculty of Dentistry, Ankara Yıldırım Beyazıt University, Ankara, Turky; ^2^Department of Endodontics, Faculty of Dentistry, Konya Necmettin Erbakan University, Konya, Turkey; ^3^Department of Endodontics, Faculty of Dentistry, Selcuk University, Konya, Turkey

**Keywords:** BioRoot RCS, Fracture resistance, GuttaFlow, One Shape

## Abstract

***Background.*** This in vitro study compared the fracture resistance of roots instrumented either with ProTaper or One Shape rotary systems and filled with one of the silicate, epoxy resin or silicone-based sealers.

***Methods.*** Sixty single-rooted extracted mandibular premolars were decoronated to a length of 13 mm and then randomly divided into two main groups (n=30) in terms of the rotary system used for preparation. Group 1 samples were instrumented with the ProTaper Universal system up to a master apical file of #F2, while samples in group 2 were enlarged with One Shape system. The two main groups were then divided into 3 subgroups in terms of the sealer used (n=10) and filled with guttapercha (either F2 or MM-GP points) of the rotary system used and one of the sealers as follows: group 1, BioRoot RCS + ProTaper F2 gutta-percha; group 2, AH Plus + ProTaper F2 gutta-percha; group 3, GuttaFlow + ProTaper F2 gutta-percha; group 4, BioRoot RCS+ MM-GP points; group 5, AH Plus + MM-GP points; and group 6, GuttaFlow + MM-GP points. Each specimen then underwent fracture testing by using a universal testing machine at a crosshead speed of 1.0 mm/min until the root fractured. Data were statistically analyzed.

***Results.*** Two-way ANOVA showed no significant differences between the groups. One Shape instruments showed significantly better fracture resistance compared to ProTaper instruments. Statistically, no significant difference was found between AHPlus, GuttaFlow and BioRoot RCS sealers.

***Conclusion.*** It can be concluded that the rotary system used for the instrumentation of teeth has some influence on the fracture resistance, while the root canal sealers do not have such an effect.

## Introduction

Endodontically treated teeth are more susceptible to vertical fractures than vital teeth. Endodontic therapy includes caries removal, access cavity preparation and root canal preparation procedures. These procedures weaken the tooth structure during endodontic therapy, dehydrate the dentin after endodontic therapy and exert excessive pressure during obturation.^1^ Vertical root fracture resistance is directly proportional to the amount of the remaining tooth structure. During root canal treatment, the possibility of vertical root fracture is higher in over-instrumented teeth.^1,2^

Nickel-titanium (NiTi) instruments have been generally used in endodontic practice because of their relatively higher reliability and better flexibility and efficiency than stainless steel files.^3^ ProTaper Universal (PTU) (Dentsply Maillefer, Ballaigues, Switzerland) is a conventionally used NiTi rotary system that operates rotationally.^3,4^ The instrument has a variable taper along its length and a convex triangular cross-section.^5^ Another system, One Shape (MicroMega, Besançon, France), is a single-file shaping system, and it is recommended that it should not be sterilized. It has two cutting edges and triple helical construction. Two cutting edges provide bending resistance, while triple helical construction is torsion-resistant. One Shape offers three different cross-sectional areas along its length for added flexibility, and the region closest to the shaft has an "S" cross-section with two cutting angles.^6^

Gutta-percha is used as the most popular root canal filling material because it has many advantages, such as easy removal from the root canal, and it is biocompatible, non-toxic and non-allergic. For hermetic seal, gutta-percha is not sufficient alone because it has no adhesion to root canal walls.^7^ Root canal sealers are needed to fill the voids between the gutta-percha cones and the voids between the gutta-percha cones and root canal walls.^8^ Lateral compaction is the most common root canal filling technique. Also, this technique prepares the ground for vertical root fracture due to the application of force to the root, and it is time-consuming.^9^ As a result of using NiTi rotary systems and with the advent of tapered gutta-percha cones, the single-cone technique has become more useful.^10^

Resin-based AH Plus root canal sealer (Dentsply, Detrey, Germany) is widely used today due to its many advantages.^11^ Another silicone-based root canal sealer is GuttaFlow (Coltene Whaledent, Langenau, Germany). GuttaFlow is a liquid filling system that combines root canal sealer and gutta-percha (GP) in a single material. GuttaFlow is biocompatible, has excellent fluidity and features a thin sealing layer.^12^ Another root canal sealer is tricalcium silicate-based BioRoot RCS (Septodont, Saint Maur-des-Fosses, France); it consists of tricalcium silicate, zirconium dioxide and povidone, water and calcium chloride.^13^ In addition, the manufacturer claims that BioRoot RCS can obturate the root canal with and without gutta-percha cones because of the excellent bonding by penetrating into the dentin structure.

This study aimed to evaluate the fracture resistance of roots instrumented either with ProTaper or One Shape rotary systems and filled with one of the silicate, epoxy resin or silicon-based sealers while the teeth were obturated either with the laterally condensed gutta-percha or the single-cone technique.

## Methods

### Specimen Preparation

Sixty extracted caries-free and single-rooted mandibular premolar teeth were decoronated to a length of 13 mm. The teeth were stored in saline solution before the experiments. The tooth lengths were determined by placing a #15 K-file (Dentsply Maillefer, Tulsa, OK) in the root canal until the apical foramen was observed and reducing the file length by 1 mm.

The teeth were randomly divided into two main groups (n=30) in terms of the instrumentation system. The samples in group 1 were instrumented with ProTaper Universal system (Dentsply Maillefer, Ballaigues, Switzerland) according to the manufacturer’s instructions; SX, S1, S2, F1 and F2 instruments were used, while the samples in group 2 were enlarged with One Shape system (Micro Mega, Besancon, France). During the instrumentation, the root canals were irrigated with 2.5 mL of 5.25% NaOCl between each change of file. After instrumentation, the specimens were irrigated with 5 mL of 17% EDTA to remove the smear layer. The root canals were dried with sterile paper points (Diadent, Diadent Group International, Burbany, BC, Canada).

Two main groups were then subdivided into three subgroups in terms of the sealer used (n=10) and filled with gutta-percha (either F2 or MM-GP points) of the rotary system used. The experimental groups were treated as follows:

**Group 1:** BioRoot RCS (Septodont, France) and ProTaper F2 gutta-percha

**Group 2:** AH Plus (Dentsply, Germany) and ProTaper F2 gutta-percha

**Group 3:** GuttaFlow (Coltene, Germany) and ProTaper F2 gutta-percha

A ProTaper F2 master gutta-percha, corresponding to the final instrument, was used as a single cone. The root canal walls were covered with sealer (BioRoot RCS, AH Plus, GuttaFlow) using paper points, and then the apical portion of the gutta-percha was coated with sealer and inserted into the root canal.

**Group 4:** BioRoot RCS, MM-GP points

**Group 5:** AH Plus, MM-GP points

**Gro up 6:** GuttaFlow, MM-GP points

An MM-GP point was used as a master cone, and the root canal walls were covered with sealer (BioRoot RCS, AH Plus, GuttaFlow) using paper points; then the apical portion of gutta-percha was coated with sealer and inserted into the root canal, followed by the placement of lateral cones for lateral condensation.

The root canal orifices were sealed with Cavit temporary filling material (3M ESPE, Germany). The obturated teeth were stored at 37ºC at 100% humidity for one week for complete setting of the sealers.

### Mechanical Testing

**Figure 1:**
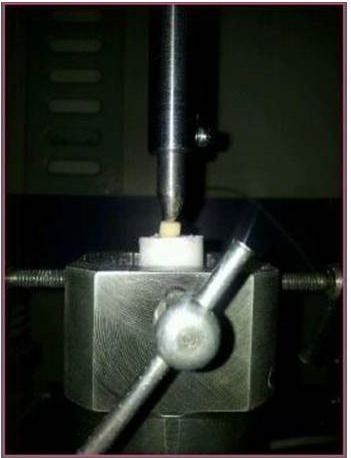
Universal testing machine (INSTRON, Llyod LRX; Lyod Instruments Ltd., Fareham, UK).

After one week, 3 mm of the roots were embedded in self-cured acrylic resin (Imicryl, Konya, Turkey) by using cylindrical molds measuring 15 mm in diameter and 13 mm in height, leaving 9 mm of the root length exposed. The temporary filling material was removed with an excavator. The specimens were mounted on the lower plate of a universal testing machine (INSTRON, Llyod LRX; Lyod Instruments Ltd., Fareham, UK) (Figure 1). A compressive loading force was applied vertically to the coronal surfaces of the roots with a loading rate of 1 mm/min until vertical root fracture (VRF) occurred. The maximum load at failure was recorded in Newton via data analysis software (Nexygen-MT, Llyod Instruments, Fareham, UK). Data were recorded and statistically analyzed with two-way ANOVA.

**Figure 2:**
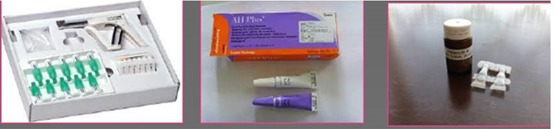
The tested sealers.

## Results

The means and standard deviations of push-out bond strengths (MPa ± SD) of the experimental groups are presented in Table 1 in terms of the sealers. Two-way ANOVA showed no significant differences between the groups (P=0.051). One Shape instruments exhibited significantly higher fracture resistance compared to ProTaper instruments (P=0.002, P<0.05). No significant differences were found between AH Plus, GuttaFlow and BioRoot RCS sealers (P=0.782, P>0.05).

**Table 1:** The means and standard deviations of push-out bond strength (MPa ± SD) in the experimental groups in terms of the sealers

**Rotary instruments**	**AH Plus** ^ 1 ^	**GuttaFlow** ^ 1 ^	**BioRoot RCS** ^ 1 ^
**One Shape** ^a^	532.60±129.63	510.50±162.38	531.70±176.44
**ProTaper Universal** ^b^	430.10±120.61	403.20±131.42	370.10±144.00

## Discussion

In this study, we compared the fracture resistance of roots instrumented with either ProTaper or One Shape rotary system and filled with one of the silicate, epoxy resin or silicone-based sealers.

In this study, no additional silicone was used as an artificial periodontal ligament to counteract the vertical force. To perfectly simulate the periodontal ligament, it is still difficult to apply a force parallel to the long axis of the tooth for proper simulation of the clinical situation.^14^ This is one of the limitations of such experiments.

The strength test is a method that has been used to examine the effect of fracture resistance of obturation materials on the root canal-filled teeth.^2,15^ Stresses generated through the root canal were transmitted along the root surface where the interfacial adhesion failed.^15^ In this study, a single load was applied to the fracture parallel to the long axis of the teeth, which produced more uniform stress distributions by using a universal test machine (Instron Corp, Canton, MA, USA).

Chemomechanical preparation of the root canal system is performed during root canal treatment to remove the infected pulp tissues; mechanical preparation should also be performed. Excessive tooth structure removal during mechanical preparation and excessive forces applied during obturation reduce the fracture resistance of root-filled teeth.^16^ Several studies have shown that decreased fracture resistance of roots after preparation with different rotary systems.^17,18^ A round cross-sectioned root canal results in more homogeneous stress distribution during root canals obturation, increasing the fracture resistance.^19^ Accordingly, ProTaper NiTi and One Shape rotary systems were used in this study to produce round-shaped root canals. One Shape instruments showed significantly better fracture resistance compared to ProTaper instruments in the present study. This could be attributed to the fact that One Shape files permit more round cross-sectional root canals during preparation because of their “S” cross-section with two cutting angles. During the root canal preparation, a relatively low concentration of NaOCl (2.5%) was used as an irrigant to minimize any adverse effects on the dentin mechanical properties.^20^

The primary goal of root canal filling is to strengthen a weakened root against fracture. To achieve an ideal and three-dimensional root canal obturation, gutta-percha cones should be used with a root canal sealer. However, root canal obturation has been known as the major reason for vertical root fracture. In the lateral condensation technique, the spreader laterally compacts the gutta-percha and adapts it to the root canal wall under consistent vertical load.^21^ However, the lateral condensation technique was used in this study because it is a widely recommended classic technique.^21^ In another study, Ersoy and Evcil^22^ investigated root canal sealers and obturation techniques.^22^ The single-cone technique showed significantly higher resistance to fracture than the lateral condensation technique. However, in this study, there were no significant differences between the root canals filled with lateral condensation technique and the other groups, which might be explained by the use of NiTi files.

Many studies have shown that epoxy resin-based sealers exhibit better adaptation with the root canal dentin compared to glass-ionomer and ZOE-based sealers.^23^ It has been shown that retention of the filling material can be mechanically improved, thereby strengthening the root canal dentin to increase the fracture resistance. The fracture resistance of AH Plus root canal sealer has already been investigated in numerous studies.^23-25^ In a previous study, AH Plus and MTA Fillapex showed significantly higher resistance to fracture than other conventional root canal sealers.^25^ With great attention to the adhesive properties and sealing ability of epoxy resin-based root canal sealers, the effect of AH Plus on the fracture resistance of root-filled teeth was compared with other types of root canal sealers in this study. In our study, there were no significant differences between AH Plus and other root canal sealers.

BioRoot RCS, commonly known as an MTA-based sealer, is a powder/liquid hydraulic tricalcium silicate-based cement recommended for the single-cone technique or cold lateral condensation root filling. It has a lower cytotoxicity than other conventional root canal sealers and might induce hard tissue deposition.^13,27^ Siboni et al^28^ showed that BioRoot RCS has high calcium ion releasing ability. A recent study showed that BioRoot RCS has a higher bioactivity than the ZOE sealer on human PDL cells.^29^ According to the manufacturer, this sealer has an integration similar to Biodentine (Septodont), trying to integrate the ideal properties of Biodentine in a root canal sealer.^30^ Also, a silicon-based sealer, GuttaFlow, has calcium ion releasing ability. In a previous study, BioRoot RCS and IRootSP resulted in higher resistance to fracture compared to MTA-Fillapex. In the present study, no significant differences were found between BioRoot RCS and the other conventional sealers. Also, in that study, the authors observed that the LTC techniques resulted in more resistance to fracture than the SC techniques, but we observed no significant differences. The differences between the results of studies might be explained by the type of the sealer used, the brand of the sealer and the experience of the practitioner.

In the present study, no differences were found in fracture resistance between the roots filled with AH Plus, BioRoot RCS and Gutta Flow and the obturation techniques. Furthermore, all the fracture patterns observed in the study after failure were irreparable.

## Conclusion

While One Shape instruments resulted in significantly better fracture resistance compared to ProTaper instruments, all the three root canal sealers examined in this study strengthened the prepared root canals with increased fracture resistance.

## Authors’ Contributions

SİY analyzed and interpreted the data and drafted and critically revised the manuscript. MA and MBA were responsible for acquisition, analysis and interpretation of data. AÜE was responsible for the study concept and design and critical revision.

## Acknowledgments

No acknowledgements.

## Funding

Not applicable.

## Competing Interests

The authors declare no competing interests with regards to the authorship and/or publication of this article.

## Ethics Approval

This study was approved by the Institutional review Board (reference no. 2018-330).
